# Additive interactions between *PRKAA1* polymorphisms and *Helicobacter pylori* CagA infection associated with gastric cancer risk in Koreans

**DOI:** 10.1002/cam4.926

**Published:** 2016-10-11

**Authors:** Sang‐Yong Eom, Seon‐Mi Hong, Dong–Hyuk Yim, Hyo‐Jin Kwon, Dae‐Hoon Kim, Hyo‐Yung Yun, Young‐Jin Song, Sei‐Jin Youn, Taisun Hyun, Joo‐Seung Park, Byung Sik Kim, Yong‐Dae Kim, Heon Kim

**Affiliations:** ^1^ Department of Preventive Medicine and Medical Research InstituteCollege of MedicineChungbuk National UniversityCheongjuKorea; ^2^ Department of SurgeryCollege of MedicineChungbuk National UniversityCheongjuKorea; ^3^ Department of Internal MedicineCollege of MedicineChungbuk National UniversityCheongjuKorea; ^4^ Department of Food and NutritionChungbuk National UniversityCheongjuKorea; ^5^ Department of SurgeryCollege of MedicineEulji UniversityDaejonKorea; ^6^ Department of SurgeryAsan Medical CenterCollege of MedicineUlsan UniversitySeoulKorea

**Keywords:** AMP‐activated protein kinase, gastric cancer, gene‐environment interaction, *Helicobacter pylori*

## Abstract

Although several studies reported genetic polymorphisms in protein kinase AMP‐activated alpha 1 catalytic subunit (*PRKAA1*) and their associations with gastric cancer risk, few have evaluated associations between *Helicobacter pylori* infection and *PRKAA1* gene‐environment interactions. Here, we evaluated the effects of interactions between *H. pylori* infection and *PRKAA1* polymorphisms on gastric cancer risk in Koreans. In this hospital‐based case–control study, *PRKAA1* genotypes were analyzed and *H. pylori* infection and CagA status were examined using a serologic method in 846 pairs of gastric cancer patients and controls matched for age and sex. *H. pylori* seropositivity was associated with a 1.43‐fold [95% confidence interval: 1.12–1.81] increase in the risk of gastric cancer, and CagA low‐positive titers during *H. pylori* infection increased the risk by 1.85‐fold (95% confidence interval, 1.38–2.48). Significant positive interaction between the *PRKAA1* rs13361707 genotype and *H. pylori* infection was verified on an additive scale [relative excess risk due to interaction, 0.55; 95% confidence interval, 0.05–1.04; *P* = 0.030], and the gene‐environment interaction between *PRKAA1* rs13361707 and CagA status was also statistically significant (relative excess risk due to interaction, 0.50; 95% confidence interval, 0.30–0.70; *P* < 0.001). Our results indicated that *H. pylori* infection, CagA status, and *PRKAA1* polymorphisms were risk factors for gastric cancer in Koreans, and that the combination of two of these factors rather than their independent effects synergistically increased the risk.

## Introduction

Gastric cancer has the fifth highest incidence rate and the third highest mortality rate worldwide. Specifically, the number of gastric cancer patients in East Asia accounts for more than half of the worldwide gastric cancer incidence and the highest incidence of gastric cancer mortality 1. Although gastric cancer incidence in Koreans has gradually decreased since 2000, it is still the most common cancer in males and the elderly (age ≥65 years). Moreover, distant metastatic gastric adenocarcinoma is a life‐threatening cancer in Koreans, with a 5‐year relative survival rate of 5.7% 2.

*Helicobacter pylori* (HP) infection is a Group 1 carcinogen 3, with many epidemiological studies demonstrating associations between chronic HP infection and gastric cancer 4. HP is a gram‐negative bacterium that proliferates in the gastric mucous membrane and has various virulence factors, including CagA, VagA, BabA, and SabA 4, 5. The serological positivity of HP infection in Koreans who have not been treated with antibiotics is 54.4%, and the HP infection rate is higher in East Asians, including Koreans, as compared with that in other races 4, 6. HP infection rates vary by race and geographic location and are affected by various factors, including age, socioeconomic level, smoking, and alcohol consumption 6, 7, 8, 9. HP infection is also affected by host genetics, which determine the development of gastric cancer acquired through interactions with HP 10, 11. According to previous meta‐analyses, the fraction of HP infection attributable to noncardia gastric cancer was ~74.7% to ~83.1% 12, 13. However, only approximately 1% of HP‐infected subjects develop gastric cancer 10. This suggests that the interaction between HP infection and host genetic factors plays a significant role in gastric carcinogenesis 11.

Genetic polymorphisms in protein kinase AMP‐activated alpha 1 catalytic subunit (*PRKAA1*) are significantly associated with gastric cancer, according to genome‐wide association study (GWAS) performed in Asians 14. Identical associations were indicated in our previous study 15 and in several other studies performed in Korean and Chinese populations 16, 17, 18, 19, and were reconfirmed in a recent GWAS performed with a European populations 20. Although the definitive role of *PRKAA1* has not yet been identified, 5′‐AMP‐activated protein kinase (AMPK) encoded by *PRKAA1* can play a significant role in carcinogenesis, as it is involved in energy metabolism, cell cycle regulation, cell proliferation, and rapamycin‐pathway inhibition 21, 22, 23. Additionally, according to recent studies, AMPK is activated by HP infection in human gastric epithelial cells, resulting in enhanced production of reactive oxygen species and inhibition of gastric epithelial cell apoptosis by HP 24, 25. However, few studies have evaluated the effects of interactions between *PRKAA1* genetic polymorphisms and HP infection on gastric cancer incidence. Therefore, we investigated these relationships in a Korean population.

## Material and Methods

### Study subjects

This study was designed as a hospital‐based case–control. Gastric cancer patients included those who were newly diagnosed with gastric cancer histologically at Chungbuk National University Hospital (*n *=* *776), Eulji University Hospital (*n *=* *776), and Asan Medical Center (*n *=* *641) from March 1997 to December 2013, for a total of 1843 patients. A control group included 1705 patients who were hospitalized for health examinations at the same hospitals during the same periods, and who had not been diagnosed with any form of cancer. A trained interviewer obtained an informed consent from all subjects recruited to the study; demographic and lifestyle information including smoking history, alcohol consumption, and diet was obtained through a structured questionnaire. Venous blood was collected from subjects from whom blood draw was possible in order to analyze their genotypes and evaluate HP infection. The collected blood was centrifuged to separate plasma and then stored at −80°C after aliquot.

Patients and controls who did not provide answers to >10% of the questionnaire (*n *=* *270), those lacking a buffy coat in their blood sample or having inadequate quality of extracted DNA (*n *=* *230), and those who had little or no serum (*n *=* *1304) were excluded from the case–control pool. Finally, a total of 846 pairs were selected by matching the patient group and the control group for age (<3 years difference) and sex at a 1:1 ratio. This study was approved by the Institutional Review Board of Chungbuk National University Hospital, Korea (IRB No. 2011‐09‐071).

### Serologic assay for HP infection and CagA status

To determine past or present HP infection, a Genedia *H. pylori* ELISA kit (Green Cross Medical Science Corp., Yongin, Korea), which was developed using Korean *H. pylori* strains, was used according to manufacturer instructions 26. Briefly, plasma HP IgG antibody was measured using 5 *μ*L plasma, and positivity was determined when the absorbance of a sample was above the cutoff value suggested by the manufacturer. Sensitivity and specificity for the HP IgG ELISA, as provided by the manufacturer, were 97.8% and 92.0%, respectively 26.

In order to evaluate CagA status, a CagA IgG antibody titer was measured using a CagA IgG ELISA Kit (Genesis Diagnostics, Alva, UK) according to manufacturer instructions, and the plasma CagA IgG antibody titer of each sample was quantified using a calibration curve. Positivity was determined when the CagA IgG antibody titer of a sample was >6.25 U/ml, Sensitivity and specificity for the CagA IgG ELISA, as provided by the manufacturer, were 96% and 97%, respectively. CagA‐positive titers were classified into two groups (low or high) based on the median CagA IgG antibody titer (12.5 U/mL) among CagA‐positive subjects.

### PRKAA1 genotyping analysis

Genomic DNA was extracted from the stored blood for analysis of *PRKAA1* genotypes. Quantitative and qualitative assessment of the extracted genomic DNA was performed using a UV spectrophotometer (NanoDrop ND‐2000, Thermo Fisher Scientific, Waltham, MA) and electrophoresis. Quality and quantity requirements for genotyping DNA samples were determined by identifying intact DNA bands after 2% agarose gel electrophoresis, and their concentrations were measured at >20 ng/*μ*L with a degree of purity >1.7. Genotype analysis was performed for the five single‐nucleotide polymorphisms (SNPs; rs10074991, rs13361707, rs154268, rs3805486, and rs6882903) associated with *PRKAA1* 14, 15, 16, 18, 19, 20, 27, 28 using a GoldenGate assay (Illumina, San Diego, CA, USA). The call rates for the examined samples and SNPs were 100%, with all five SNPs meeting the Hardy–Weinberg equilibrium in the control group.

### Statistical analysis

In order to compare general characteristics between the patient and control groups, a Student's *t*‐test was performed for continuous variables [e.g., age, height, weight, and body mass index (BMI)], and a chi‐square test and conditional logistic regression was performed for categorical variables (e.g., smoking, drinking, and education level) and categorized variables (cumulative smoking amount and weekly alcohol intake). The variables, such as smoking, alcohol intake, education level, and BMI, that showed significant differences between the patient and control groups or were found to have significant associations with gastric cancer were considered covariates and included in the final multivariate model for analysis.

Multivariate conditional logistic regression analysis was performed in order to evaluate the effects of HP infection, CagA status, and *PRKAA1* SNPs on gastric cancer incidence, and the results were presented as crude and adjusted odds ratios (ORs) and 95% confidence intervals (CIs). The Benjamini–Hochberg procedure was performed for multiple test comparison correction 29, and false discovery rate (FDR)‐adjusted *P* values were suggested.

Gene‐environmental interactions in gastric cancer were tested on additive and multiplicative scales. Additive interactions were evaluated by estimating relative excess risk due to interaction (RERI), and significant additive interactions were defined when RERI was >0 and the 95% CI did not include 0 30. Multiplicative interactions were evaluated using the product term of two factors, with the ratio of ORs suggested as an indicator 31. In cases where information about pathophysiological characteristics for the gastric cancer patients included the location of the gastric cancer and histological characteristics, subgroup analysis regarding those pathophysiological characteristics was performed using the same method. All statistical analyses were performed using SAS version 9.2 (SAS Institute, Cary, NC).

## Results

### General characteristics of the study subjects

There were no differences in the mean age and sex distributions between gastric cancer patients and the control group, given that they were matched by age and sex. Current or former smokers exhibited a 1.4‐fold greater risk of developing gastric cancer as compared with nonsmokers, and smokers with >40 pack‐years of cumulative smoking exhibited a 1.8‐fold greater risk relative to nonsmokers. Alcohol drinkers whose weekly intake was >280 g exhibited a 1.6‐fold increase in risk relative to nondrinkers. The education level of gastric cancer patients was significantly lower than that of the control group, and the mean BMI was also significantly different (Table 1).

**Table 1:** General characteristics of the study population

Variables	Cases	Controls	*P* or OR^a^ (95% CI)
*n*	846	846	
Age, mean ± SD	56.9 ± 10.8	56.3 ± 10.9	0.272
Gender, *n* (%)			
Males	544 (64.3)	544 (64.3)	
Females	302 (35.7)	302 (35.7)	
Smoking status, *n* (%)			
Nonsmokers	345 (40.8)	380 (44.9)	1.00 (ref.)
Smokers	501 (59.2)	466 (55.1)	1.43 (1.08, 1.90)
Cumulative smoking amount, *n* (%)			
Nonsmokers	345 (40.8)	380 (44.9)	1.00 (ref.)
0–19 pack‐years	126 (14.9)	123 (14.5)	1.34 (0.95, 1.90)
20–39 pack‐years	193 (22.8)	206 (24.4)	1.23 (0.89, 1.70)
>40 pack‐years	182 (21.5)	137 (16.2)	1.79 (1.27, 2.54)
Alcohol intake amount, g/week, *n* (%)			
Nondrinkers	322 (38.1)	327 (38.7)	1.00 (ref.)
>0 to ≤280 g/week	318 (37.6)	374 (44.2)	0.89 (0.70, 1.15)
>280 g/week	206 (24.4)	145 (17.1)	1.58 (1.15, 2.17)
Education level, *n* (%)			
< High school	503 (61.0)	354 (42.5)	1.00 (ref.)
≥ High school	321 (39.0)	480 (57.6)	0.35 (0.28, 0.46)
Height, cm	162.9 ± 8.8	163.7 ± 8.3	0.083
Weight, kg	58.7 ± 11.0	63.3 ± 10.4	<0.001
Body mass index, kg/m^2^	22.4 ± 3.1	23.5 ± 2.9	<0.001

SD, standard deviation; CI, confidence interval; OR, odds ratio.

a Conditional logistic regression analysis.

### HP infection and gastric cancer

There was a higher degree of HP IgG antibody positivity in gastric cancer patients than in the control group (77.4% vs. 70.6%), and HP‐infected individuals showed a 1.43‐fold (95% CI: 1.12–1.81) increase in the risk of gastric cancer when compared with HP‐negative individuals. CagA positivity was also higher in the patient group (59.0%) relative to the control group (55.6%), but CagA status was not associated with increased risk of gastric cancer (OR, 1.15; 95% CI: 0.92–1.43). However, when compared with HP‐negative individuals who were CagA negative, HP‐infected individuals who were CagA positive had a significantly increased risk of gastric cancer (OR, 1.37; 95% CI: 1.07–1.76). Furthermore, when stratified against CagA seropositivity according to the antibody titer, HP‐infected subjects with low‐positive CagA titer (6.25–12.5 U/mL) had an increased risk of gastric cancer compared to HP‐negative subjects who were CagA negative (OR, 1.85; 95% CI: 1.38–2.48) (Table 2).

**Table 2:** Distributions of *Helicobacter pylori* infection and CagA status, and risk of gastric cancer

Variable	*n* (%)		COR (95% CI)	AOR (95% CI)
	Cases	Controls		
*H. pylori* infection				
Negative	191 (22.6)	249 (29.4)	1.00 (ref.)	1.00 (ref.)
Positive	655 (77.4)	597 (70.6)	1.41 (1.14, 1.76)	1.43 (1.12, 1.81)
CagA status^a^				
Negative	344 (41.0)	370 (44.4)	1.00 (ref.)	1.00 (ref.)
Positive	495 (59.0)	464 (55.6)	1.15 (0.95, 1.40)	1.15 (0.92, 1.43)
*H. pylori* and CagA status				
*H. pylori* negative/CagA negative	191 (22.8)	249 (29.9)	1.00 (ref.)	1.00 (ref.)
*H. pylori* positive/CagA negative	153 (18.1)	121 (14.5)	1.64 (1.20, 2.25)	1.68 (1.19, 2.39)
*H. pylori* positive/CagA positive	495 (59.1)	464 (55.6)	1.37 (1.09, 1.71)	1.37 (1.07, 1.76)
* H. pylori* positive/CagA low^b^ positive	272 (32.5)	176 (21.1)	1.94 (1.49, 2.54)	1.85 (1.38, 2.48)
* H. pylori* positive/CagA high^c^ positive	223 (26.6)	288 (34.5)	0.96 (0.74, 1.26)	1.03 (0.77, 1.37)

AOR, adjusted odds ratio (adjusted for cumulative smoking amount, alcohol intake amount, body mass index, and education level); COR, crude odds ratio; CI, confidence interval.

a There are missing data of CagA status for 7 gastric cancer cases and 12 controls.

b Defined as CagA IgG antibody titer <12.5 U/mL among the CagA‐positive results.

c Defined as CagA IgG antibody titer ≥12.5 U/mL among the CagA‐positive results.

### PRKAA1 polymorphisms and gastric cancer

Genotype distributions of the five *PRKAA1* SNPs are presented in Table 3. Individuals with at least one variant allele of *PRKAA1* rs10074991 or rs13361707 had a significantly increased risk of gastric cancer compared to the carriers with the homozygous wild‐type allele. For the variant homozygote of these two SNPs, the risk of gastric cancer was 2.23‐ and 2.20‐fold higher relative to that of the wild‐type homozygote, respectively. The statistical significance remained after correction of multiple comparison effects 32. Interestingly, although *PRKAA1* rs154268 and rs6882903 heterozygotes increased the risk of gastric cancer by 1.52‐ and 1.34‐fold compared with their wild‐type homozygote, respectively, there was no significant association observed for the variant homozygote.

**Table 3:** Associations between five genetic polymorphisms of *PRKAA1* and risk of gastric cancer

SNP ID	Genotypes	Cases, *n* (%)	Controls, *n* (%)	COR (95% CI)	*P*	AOR (95% CI)	*P*	FDR^a^
rs10074991	A/A	177 (20.9)	248 (29.3)	1.00 (ref.)		1.00 (ref.)		
	A/G	421 (49.8)	429 (50.7)	1.40 (1.11, 1.78)	0.005	1.42 (1.08, 1.85)	0.011	0.018
	G/G	248 (29.3)	169 (20.0)	2.12 (1.59, 2.81)	2.43 × 10^‐7^	2.23 (1.62, 3.09)	1.08 × 10^‐6^	1.19 × 10^‐5^
	A/G+G/G	669 (79.1)	598 (70.7)	1.58 (1.26, 1.98)	7.70 × 10^‐5^	1.62 (1.25, 2.09)	2.49 × 10^‐4^	0.001
rs13361707	T/T	176 (20.8)	248 (29.3)	1.00 (ref.)		1.00 (ref.)		
	T/C	421 (49.8)	424 (50.1)	1.45 (1.15, 1.85)	0.003	1.44 (1.10, 1.88)	0.007	0.016
	C/C	249 (29.4)	174 (20.6)	2.08 (1.56, 2.78)	4.34 × 10^‐7^	2.20 (1.59, 3.04)	1.58 × 10^‐6^	1.19 × 10^‐5^
	T/C+C/C	670 (79.2)	598 (70.7)	1.58 (1.27, 1.99)	6.28 × 10^‐5^	1.86 (1.40, 2.48)	1.98 × 10^‐4^	0.001
rs154268	T/T	466 (55.1)	545 (64.4)	1.00 (ref.)		1.00 (ref.)		
	T/C	335 (39.6)	270 (31.9)	1.44 (1.18, 1.76)	4.00 × 10^‐4^	1.52 (1.21, 1.91)	3.00 × 10^‐4^	0.001
	C/C	45 (5.3)	31 (3.7)	1.73 (1.07, 2.80)	0.025	1.53 (0.90, 2.62)	0.119	0.149
	T/C+C/C	380 (44.9)	301 (35.6)	1.47 (1.21, 1.78)	1.00 × 10^‐4^	1.52 (1.22, 1.90)	2.00 × 10^‐4^	0.001
rs3805486	T/T	476 (56.3)	424 (50.1)	1.00 (ref.)		1.00 (ref.)		
	T/C	329 (38.9)	362 (42.8)	0.81 (0.67, 0.99)	0.038	0.85 (0.68, 1.05)	0.135	0.150
	C/C	41 (4.9)	60 (7.1)	0.61 (0.40, 0.93)	0.020	0.71 (0.45, 1.12)	0.140	0.150
	T/C+C/C	370 (43.7)	422 (49.9)	0.79 (0.65, 0.95)	0.013	0.83 (0.67, 1.02)	0.082	0.112
rs6882903	C/C	548 (64.8)	600 (70.9)	1.00 (ref.)		1.00 (ref.)		
	C/A	271 (32.0)	226 (26.7)	1.30 (1.06, 1.61)	0.013	1.34 (1.06, 1.70)	0.016	0.023
	A/A	27 (3.2)	20 (2.4)	1.46 (0.81, 2.65)	0.211	1.39 (0.71, 2.72)	0.340	0.340
	C/A+A/A	298 (35.2)	246 (29.1)	1.32 (1.08, 1.61)	0.008	1.34 (1.07, 1.69)	0.011	0.018

AOR, adjusted odds ratio (adjusted for cumulative smoking amount, alcohol intake amount, body mass index, and education level); COR, crude odds ratio; FDR, false discovery rate (adjusted using the Benjamini–Hochberg procedure); CI, confidence interval.

a FDR‐adjusted *P*‐value.

### Gene‐environmental interaction between PRKAA1 genotypes and HP infection on gastric cancer risk

In order to evaluate the effects of the gene‐environmental interaction between *PRKAA1* polymorphisms and HP infection on gastric cancer incidence, analysis of their independent and joint effects was performed (Table 4). The OR for the combination for *PRKAA1* rs10074991 A/G+G/G genotype and positive HP infection (OR, 1.87; 95% CI: 1.21–2.10) was greater than the ORs for the *PRKAA1* rs10074991 A/G+G/G genotype alone (OR, 1.17; 95% CI: 0.73–1.88) and positive HP infection alone (OR, 1.17; 95% CI: 0.73–1.87). The additive interaction between the *PRKAA1* rs10074991 genotype and HP infection in relation to gastric cancer risk was statistically significant (RERI, 0.53; 95% CI: 0.03–1.03; *P* = 0.037), while the multiplicative interaction was not statistically significant (OR, 1.36; 95% CI: 0.79–2.34; *P* = 0.261). Similarly, the interaction between the *PRKAA1* rs13361707 genotype and HP infection was statistically significant only on an additive scale (RERI, 0.55; 95% CI: 0.05–1.04; *P* = 0.030). Genetic‐ and gene‐environmental effects of *PRKAA1* rs10074997 and rs13361707 on the risk of gastric cancer were almost identical. This identical pattern can be explained by the strong linkage disequilibrium between the two SNPs 15. In the evaluation of gene‐environment interactions between the *PRKAA1* rs13361707 genotype and CagA status of HP infection as related to gastric cancer incidence, the RERI was 0.50 (95% CI: 0.30–0.70; *P* < 0.001), indicating a significant interaction (Table 5). The remaining three SNPs (rs154268, rs3805486, and rs6882903) did not show statistically significant interactions with HP CagA status.

**Table 4:** Effects of interactions between HP infection and five genetic polymorphisms of *PRKAA1* on the risk of gastric cancer

SNP ID	Genotypes	HP infection status				ORs^a^ (95% CI) for HP‐positive within strata of genotype	RERI^b^ (95% CI)	Ratio of ORs^c^ (95% CI)
		Negative		Positive				
		No. of cases/controls	OR^a^ (95% CI)	No. of cases/controls	OR^a^ (95% CI)			
rs10074991	A/A	42/63	1.00 (ref.)	135/185	1.17 (0.73, 1.88)	1.17 (0.73, 1.88)	0.53 (0.03, 1.03)	1.36 (0.79, 2.34)
	A/G+G/G	149/186	1.17 (0.73, 1.87)	520/412	1.87 (1.21, 2.88)	1.59 (1.22, 2.08)	*P* = 0.037	*P* = 0.261
	ORs^a^ (95% CI) for genotype within strata of HP infection status		1.17 (0.73, 1.87)		1.60 (1.22, 2.10)			
rs13361707	T/T	42/63	1.00 (ref.)	134/185	1.16 (0.72, 1.87)	1.16 (0.72, 1.87)	0.55 (0.05, 1.04)	1.38 (0.80, 2.37)
	T/C+C/C	149/186	1.17 (0.73, 1.87)	521/412	1.88 (1.22, 2.89)	1.60 (1.23, 2.08)	*P* = 0.030	*P* *=* 0.243
	ORs^a^ (95% CI) for genotype within strata of HP infection status		1.17 (0.73, 1.87)		1.62 (1.24, 2.12)			
rs154268	T/T	100/156	1.00 (ref.)	366/389	1.58 (1.17, 2.14)	1.58 (1.17, 2.14)	0.01 (−0.72, 0.74)	0.87 (0.54, 1.39)
	T/C+C/C	91/93	1.59 (1.06, 2.39)	289/208	2.18 (1.58, 3.02)	1.38 (0.96, 1.97)	*P* = 0.983	*P* *=* 0.549
	ORs^a^ (95% CI) for genotype within strata of HP infection status		1.59 (1.06, 2.39)		1.38 (1.09, 1.76)			
rs3805486	T/T	112/132	1.00 (ref.)	364/292	1.56 (1.14, 2.13)	1.56 (1.14, 2.13)	−0.20 (−0.72, 0.33)	0.91 (0.57, 1.44)
	T/C+C/C	79/117	0.87 (0.58, 1.30)	291/305	1.22 (0.89, 1.68)	1.40 (0.99, 1.97)	*P* = 0.458	*P = *0.680
	ORs^a^ (95% CI) for genotype within strata of HP infection status		0.87 (0.58, 1.30)		0.79 (0.62, 1.00)			
rs6882903	C/C	116/173	1.00 (ref.)	432/427	1.63 (1.23, 2.17)	1.63 (1.23, 2.17)	−0.24 (−1.01, 0.53)	0.77 (0.47, 1.25)
	C/A+A/A	75/76	1.56 (1.03, 2.37)	223/170	1.95 (1.41, 2.70)	1.29 (0.87, 1.92)	*P *=* *0.543	*P *=* *0.289
	ORs^a^ (95% CI) for genotype within strata of HP infection status		1.56 (1.03, 2.37)		1.20 (0.94, 1.55)			

CI, confidence intervals; HP*, Helicobacter pylori*; OR, odds ratios; RERI, relative excess risk due to interaction

a ORs were adjusted for age, sex, cumulative smoking amount, alcohol intake amount, body mass index, and education level.

b RERI > 0 indicates an additive interaction.

c Ratio of ORs measures the interactions on a multiplicative scale.

**Table 5:** Effects of interactions between HP infection, CagA status, and *PRKAA1* rs13361707 polymorphisms on the risk of gastric cancer

rs13361707 genotype	HP infection/CagA status			ORs^a^ (95% CI) for positive/negative within strata of genotype	ORs^a^ (95% CI) for both positive within strata of genotype
	Both negative	Positive/negative	Both positive		
	OR^a^ (95% CI)	OR^a^ (95% CI)	OR^a^ (95% CI)		
T/T	1.00 (ref.)	1.21 (0.63, 2.33)	1.13 (0.71, 1.78)	1.21 (0.63, 2.33)	1.13 (0.71, 1.78)
T/C+C/C	1.16 (0.75, 1.81)	2.20 (1.37, 3.54)	1.81 (1.21, 2.72)	1.90 (1.31, 2.75)	1.56 (1.18, 2.05)
ORs^a^ (95% CI) for genotype within strata of HP infection status	1.16 (0.75, 1.81)	1.83 (0.97, 3.44)	1.15 (0.71, 1.86)		
RERI^b^ (95% CI)		0.83 (−0.12, 1.77)	0.50 (0.30, 0.70)		
		*P* = 0.087	*P* < 0.001		
Ratio of ORs^c^ (95% CI)		1.54 (0.71, 3.31)	1.38 (0.79, 2.41)		
		*P* = 0.274	*P* = 0.261		

OR, odds ratio; RERI, relative excess risk due to interaction.

a ORs were adjusted for age, sex, cumulative smoking amount, alcohol intake amount, body mass index, and education level.

b RERI > 0 indicates an additive interaction.

c Ratio of ORs measures the interactions on a multiplicative scale.

Subgroup analysis was conducted on 308 gastric cancer patients based on anatomic location of cancer and histologic type. In subgroup analysis, we also evaluated the different effects of interactions between the *PRKAA1* rs13361707 genotype and HP infection on gastric cancer incidence according to pathophysiological characteristics. Based on cancer location, additive interaction between the *PRKAA1* rs13361707 genotype and HP infection was shown to be significant in cardia gastric cancer (RERI, 1.25; 95% CI: 0.08–2.41; *P* = 0.036). Similarly, positive additive interaction was observed in noncardia gastric cancer, although with borderline significance (RERI, 0.66; 95% CI: −0.11–1.43; *P* = 0.092). However, comparisons based on histology indicated significant interactions between the *PRKAA1* rs13361707 genotype and HP infection only in intestinal type (RERI, 0.85; 95% CI: 0.24–1.47; *P* = 0.007), and not in diffuse type (RERI, 0.96; 95% CI: −1.01–2.93; *P* = 0.338) (Table S1).

## Discussion

This study showed that HP infection, CagA status, and *PRKAA1* polymorphisms are risk factors for gastric cancer in Koreans; specifically, the additive interaction between two of these factors increases gastric cancer risk. This study is the first to verify that CagA virulence factor‐related HP infection and *PRKAA1* polymorphisms have significant effects on gastric cancer risk through the gene‐environment interactions.

Previous studies have reported that the interaction between the genetic factors of a host and HP infection plays a significant role in gastric cancer carcinogenesis. The SNPs identified in previous studies were located on HP‐related genes (*IL‐1B, IL‐10, IL‐17, PGC, PTPN1, NOD1, NOD2*), carcinogen detoxification‐related gene (*GSTP1*), and cell proliferation‐related gene (*COX2*) 17, 32, 33, 34, 35, 36, 37, 38. In addition, DNA repair‐related genes, pattern‐recognition receptors, and genes identified in GWASs (*PSCA* and *MUC1*) have important roles in gastric cancer carcinogenesis 39, 40, 41. These genes may be involved in various carcinogenic steps, such as initiation, promotion, and progression of gastric cancer. Recently, Kodaman et al. reported that the interaction between genetic ancestries of humans and HP bacteria affects gastric cancer risk, with the risk increasing when in case of genetic mismatch, and decreasing in case of genetic matches 42. This phenomenon was explained to be a result of genetic co‐evolution of humans and HP. Although the HP infection rate in Koreans is relatively high as compared to that in other races 6, the risk (pooled relative risk: 1.69) of HP‐related gastric cancer in studies performed in Koreans was not higher than that observed in other races (pooled relative risk: 5.9) 12, 43. This implies that HP infection is not a strong independent risk factor for gastric cancer, and that the various and complicated interactions with genetic factors strengthen its carcinogenic potency. *PRKAA1‐*related mechanisms associated with gastric carcinogenesis remain unknown. However, several studies consistently reported associations between *PRKAA1* rs13361707 and gastric cancer 14, 15, 17, 18, 19, 20. Additionally, a recent meta‐analysis concerning *PRKAA1* polymorphisms found that the *PRKAA1* rs13361707 C allele was strongly associated with risk of noncardia gastric cancer 28. *PRKAA1* rs13361707 is located in intron 1 of the *PRKAA1* gene, and the exact function of the SNP has not been determined. *PRKAA1* amplification and genetic alteration occur simultaneously along with other oncogenes, such as KRAS, in various solid tumors including gastric cancer 44. Interestingly, *PRKAA1* plays dual roles in carcinogenesis, both as a tumor suppressor that controls mTOR activation and as an oncogene involved in protection of cancer cell viability by maintaining NADPH and ATP levels under metabolic stress conditions 44, 45, 46. Furthermore, AMPK is activated by HP infection in gastric epithelial cells, resulting in inhibition of ROS production and apoptosis in gastric epithelial cells 24, 25. These findings supported that interactions between *PRKAA1* polymorphisms and HP infection play significant roles in gastric carcinogenesis, like presented in this study.

This study also verified that CagA status affects gastric cancer risk through its interaction with the *PRKAA1* rs13361707 SNP. Although no studies specifically evaluating this interaction exist, a recent report predicted the biological association between these two entities. HP CagA is involved in carcinogenesis by depleting glycogen synthase kinase 3 (GSK3), thereby inducing Snail‐mediated epithelial–mesenchymal transition 47. Interestingly, GSK3 interacts with and inhibits AMPK under anabolic conditions 48.

In this study, gene‐environmental interactions between *PRKAA1* SNPs and HP infection were evaluated on additive and multiplicative scales, resulting in one significantly positive additive interaction. Similar to our results, Li et al. reported additive interactions between the *NOD2* rs7189226 and HP infection; however, few studies involving a similar design have reported results of interactions on an additive scale. Knol and VanderWeele recommended that all results, both additive and multiplicative in nature, be presented in a study concerning gene‐environmental interactions 31, given that additive‐scale interactions are important for the evaluation of public health interventions. Therefore, SNPs involved in additive interactions, including those involving *PRKAA1* rs13361707, should be considered in the evaluation of cost‐effective HP‐eradication therapies.

In subgroup analyses by cancer location, a significant positive additive interaction between the *PRKAA1* rs13361707 genotype and HP infection was observed in cardia gastric cancer (RERI, 1.25; 95% CI: 0.08–2.41; *P* = 0.036), whereas borderline significance was observed in noncardia gastric cancer (RERI, 0.66; 95% CI: −0.11–1.43; *P* = 0.092). However, the pattern of additive interaction was similar (RERI > 0) in both noncardia and cardia gastric cancer. Similarly, a previous Korean study indicated that the association between *PRKAA1* rs13361707 and gastric cancer risk did not vary depending on the tumor site 18. Shakeri et al. observed that HP CagA infection was associated with a significantly increased risk not only for cardia, but for noncardia gastric adenocarcinoma 49. In contrast, several studies reported that the association between *PRKAA1* rs13361707 and gastric cancer risk is stronger in noncardia gastric cancer than in cardia cancer 14, 17. A comparison based on histological type showed significant additive interactions only in intestinal‐type cancer; consistent with our results, Hwang et al. also indicated that the *PRKAA1* SNP was more strongly associated with intestinal‐type gastric cancer than diffuse‐type gastric cancer 16. In addition, intestinal‐type gastric cancer is associated more with HP infection when compared to diffuse‐type gastric cancer 50, since stratified analysis for interaction between the *PRKAA1* rs13361707 SNP and HP infection according to the anatomic location of cancer and histological type of cancer was considerably low.

In this study, HP infection was associated with an increase in the risk of gastric cancer, which was highest in subjects with a low‐positive CagA antibody titer. Similar to our findings, two Japanese cohort studies also reported that a low‐seropositive antibody titer for HP or CagA was associated with an increased incidence of gastric cancer 51, 52. The HP IgG antibody titer reduced and disappeared with the progression from severe gastric mucosal atrophy to gastric cancer 53. High‐positive antibody titers for HP have been observed in individuals with active inflammation, and low‐positive titers in those with progressive atrophy of the gastric mucosa, respectively 54. Hatakeyama has suggested in a “CagA‐mediated hit‐and‐run carcinogenesis” model that CagA‐related HP infection was significantly associated with the initiation of gastric carcinogenesis, but the maintenance and progression of gastric cancer was not dependent upon CagA status 55.

This study has some limitations. First, it was impossible to measure the cancer patients' actual HP infection status prior to gastric cancer development because of our retrospective study design. Second, we did not assess information of HP eradication treatment for both cases and controls group. Risk factors for HP infection in Koreans include being male, of advanced age, and having a low socioeconomic status, all of which are related to hygiene conditions, immune status, and low possibility of HP‐eradication therapy 6, 56. Therefore, this study controlled for these issues by matching and adjusting for each factor. Because gastric cancer patient's serum samples were collected at the time of diagnosis, it is possible that serological test results become negative as the environment associated with the gastric mucous membrane changes into an environment unsuitable for HP proliferation during gastric cancer progression 43. Therefore, we cannot rule out the possibility that the HP seropositivity rates may have been underestimated especially in gastric cancer patients. However, this misclassification might lead to our results toward the null; nevertheless, we observed the significant independent and interactive association of HP infection and *PRKAA1* polymorphisms on gastric cancer risk.

In conclusion, this hospital‐based case–control study showed that the additive interaction between HP infection and *PRKAA1* polymorphisms are significantly associated with gastric cancer risk in Koreans. These findings support that the gene‐environmental interaction between HP infection and host genetic factors plays a pivotal role in gastric carcinogenesis.

## Conflicts of Interest

None declared.

## Supporting information


**Table S1**. Effects of interactions between HP infection and five *PRKAA1* polymorphisms on the risk of gastric cancer according to covariates.
