# Supplementary material for: Additive interactions between PRKAA1 polymorphisms and Helicobacter pylori CagA infection associated with gastric cancer risk in Koreans
Source: Cancer Med. 2016 Oct 11;5(11):3236–335. doi: 10.1002/cam4.926 (PMC5119980; doi:10.1002/cam4.926)
Supplement: Supplementary file 1 — Table S1. Effects of interactions between HP infection and five PRKAA1 polymorphisms on the risk of gastric cancer according to covariates. [file CAM4-5-3236-s001.docx]

**Supplementary Table 1**. Effects of interactions between HP infection and five *PRKAA1* polymorphisms on the risk of gastric cancer according to covariates.

| Variables | | rs13361707 genotypes | HP infection status | | ORs (95% CI)^a^ for HP positive within strata of genotype | RERI (95% CI)^b^ | Ratio of ORs (95% CI)^c^ |
| --- | --- | --- | --- | --- | --- | --- | --- |
|  |  |  | Negative | Positive |  |  |  |
|  |  |  | OR (95% CI)^a^ | OR (95% CI)^a^ |  |  |  |
| Age | < 56.3 years | T/T | 1.00 (ref.) | 1.59 (0.77, 3.26) | 1.62 (0.79, 3.31) | 1.29 (0.40, 2.18) | 1.75 (0.76, 1.39) |
|  |  | T/C+C/C | 1.06 (0.50, 2.23) | 2.94 (1.5, 5.74) | 2.83 (1.84, 4.36) | *P*=0.004 | P=0.191 |
|  |  | ORs (95% CI)^a^ for genotype within strata of HP infection status | 1.14 (0.54, 2.4) | 1.84 (1.26, 2.70) |  |  |  |
|  | ≥ 56.3 years | T/T | 1.00 (ref.) | 0.97 (0.51, 1.84) | 1.05 (0.54, 2.05) | 0.18 (−0.52, 0.89) | 1.17 (0.56, 2.44) |
|  |  | T/C+C/C | 1.19 (0.64, 2.23) | 1.34 (0.75, 2.40) | 1.13 (0.80, 1.60) | P=0.609 | P*=*0.683 |
|  |  | ORs (95% CI)^a^ for genotype within strata of HP infection status | 1.16 (0.61, 2.22) | 1.39 (0.94, 2.04) |  |  |  |
|  |  |  |  |  |  |  |  |
| Sex | Males | T/T | 1.00 (ref.) | 0.85 (0.47, 1.56) | 0.83 (0.45, 1.54) | 0.57 (0.07, 1.06) | 1.69 (0.85, 3.35) |
|  |  | T/C+C/C | 0.96 (0.53, 1.75) | 1.38 (0.80, 2.40) | 1.44 (1.04, 2.00) | P=0.023 | P*=*0.134 |
|  |  | ORs (95% CI)^a^ for genotype within strata of HP infection status | 0.95 (0.53, 1.78) | 1.62 (1.15, 2.28) |  |  |  |
|  | Females | T/T | 1.00 (ref.) | 1.89 (0.87, 4.11) | 2.06 (0.91, 4.68) | 0.64 (−0.54, 1.83) | 1.06 (0.43, 2.59) |
|  |  | T/C+C/C | 1.53 (0.70, 3.32) | 3.06 (1.50, 6.24) | 1.95 (1.24, 3.07) | P=0.291 | P*=*0.902 |
|  |  | ORs (95% CI)^a^ for genotype within strata of HP infection status | 2.10 (0.90, 4.90) | 1.64 (1.06, 2.54) |  |  |  |
|  |  |  |  |  |  |  |  |
| BMI | < 23.5 | T/T | 1.00 (ref.) | 1.01 (0.52, 1.98) | 1.02 (0.52, 2.00) | 0.83 (0.24, 1.41) | 1.83 (0.85, 3.94) |
|  |  | T/C+C/C | 1.02 (0.53, 1.98) | 1.87 (1.01, 3.46) | 1.81 (1.26, 2.61) | P=0.005 | P*=*0.125 |
|  |  | ORs (95% CI)^a^ for genotype within strata of HP infection status | 0.98 (0.50, 1.91) | 1.87 (1.28, 2.71) |  |  |  |
|  | ≥ 23.5 | T/T | 1.00 (ref.) | 1.26 (0.65, 2.46) | 1.31 (0.66, 2.60) | 0.27 (−0.54, 1.09) | 1.09 (0.51, 2.36) |
|  |  | T/C+C/C | 1.31 (0.68, 2.55) | 1.85 (1.01, 3.39) | 1.40 (0.96, 2.05) | P=0.515 | P*=*0.825 |
|  |  | ORs (95% CI)^a^ for genotype within strata of HP infection status | 1.43 (0.72, 2.86) | 1.46 (0.99, 2.16) |  |  |  |
|  |  |  |  |  |  |  |  |
| Smoking | Non-smokers | T/T | 1.00 (ref.) | 1.39 (0.70, 2.78) | 1.46 (0.72, 2.97) | 0.43 (−0.45, 1.33) | 1.14 (0.51, 2.55) |
|  |  | T/C+C/C | 1.38 (0.69, 2.78) | 2.21 (1.17, 4.18) | 1.59 (1.05, 2.42) | P=0.338 | P*=*0.751 |
|  |  | ORs (95% CI)^a^ for genotype within strata of HP infection status | 1.65 (0.78, 3.46) | 1.58 (1.07, 2.34) |  |  |  |
|  | Smokers | T/T | 1.00 (ref.) | 0.98 (0.51, 1.88) | 0.97 (0.50, 1.89) | 0.67 (0.10, 1.23) | 1.66 (0.79, 3.46) |
|  |  | T/C+C/C | 1.02 (0.54, 1.94) | 1.66 (0.92, 3.02) | 1.62 (1.15, 2.29) | P=0.020 | P*=*0.180 |
|  |  | ORs (95% CI)^a^ for genotype within strata of HP infection status | 1.00 (0.52, 1.91) | 1.72 (1.18, 2.51) |  |  |  |
|  |  |  |  |  |  |  |  |
| Drinking | Non-drinker | T/T | 1.00 (ref.) | 1.57 (0.73, 3.36) | 1.69 (0.77, 3.73) | 0.41 (−0.59, 1.42) | 1.11 (0.47, 2.64) |
|  |  | T/C+C/C | 1.33 (0.63, 2.79) | 2.31 (1.16, 4.58) | 1.73 (1.13, 2.64) | P=0.418 | P*=*0.814 |
|  |  | ORs (95% CI)^a^ for genotype within strata of HP infection status | 1.44 (0.66, 3.15) | 1.48 (0.94, 2.33) |  |  |  |
|  | Drinkers | T/T | 1.00 (ref.) | 0.94 (0.51, 1.74) | 0.91 (0.49, 1.70) | 0.59 (0.06, 1.12) | 1.72 (0.85, 3.48) |
|  |  | T/C+C/C | 1.01 (0.55, 1.87) | 1.55 (0.88, 2.73) | 1.54 (1.10, 2.17) | P=0.028 | P*=*0.135 |
|  |  | ORs (95% CI)^a^ for genotype within strata of HP infection status | 1.00 (0.54, 1.88) | 1.66 (1.18, 2.33) |  |  |  |
|  |  |  |  |  |  |  |  |
| Education level | <High school | T/T | 1.00 (ref.) | 1.21 (0.64, 2.31) | 1.25 (0.65, 2.41) | 0.32 (−0.42, 1.07) | 1.18 (0.57, 2.46) |
|  |  | T/C+C/C | 1.22 (0.66, 2.29) | 1.76 (0.99, 3.13) | 1.43 (0.99, 2.04) | P=0.395 | P*=*0.660 |
|  |  | ORs (95% CI)^a^ for genotype within strata of HP infection status | 1.31 (0.69, 2.51) | 1.43 (0.97, 2.13) |  |  |  |
|  | ≥High school | T/T | 1.00 (ref.) | 1.11 (0.54, 2.27) | 1.12 (0.54, 2.31) | 0.79 (0.13, 1.46) | 1.67 (0.74, 3.76) |
|  |  | T/C+C/C | 1.11 (0.54, 2.28) | 2.01 (1.03, 3.91) | 1.84 (1.24, 2.74) | P=0.019 | P*=*0.220 |
|  |  | ORs (95% CI)^a^ for genotype within strata of HP infection status | 1.23 (0.58, 2.62) | 1.84 (1.26, 2.68) |  |  |  |
|  |  |  |  |  |  |  |  |
| Cancer location | Cardia (n=162) | T/T | 1.00 (ref.) | 0.57 (0.1, 3.30) | 0.51 (0.08, 3.11) | 1.25 (0.08, 2.41) | 3.54 (0.49, 25.86) |
|  |  | T/C+C/C | 0.80 (0.15, 4.18) | 1.62 (0.37, 7.17) | 2.05 (0.81, 5.21) | P=0.036 | P*=*0.212 |
|  |  | ORs (95% CI)^a^ for genotype within strata of HP infection status | 0.53 (0.08, 3.57) | 2.74 (0.92, 8.13) |  |  |  |
|  | Non-cardia (n=246) | T/T | 1.00 (ref.) | 1.22 (0.57, 2.57) | 1.34 (0.63, 2.85) | 0.66 (−0.11, 1.43) | 1.39 (0.60, 3.22) |
|  |  | T/C+C/C | 1.28 (0.61, 2.67) | 2.15 (1.09, 4.25) | 1.71 (1.16, 2.52) | P=0.092 | P*=*0.447 |
|  |  | ORs (95% CI)^a^ for genotype within strata of HP infection status | 1.23 (0.58, 2.63) | 1.77 (1.18, 2.66) |  |  |  |
|  |  |  |  |  |  |  |  |
| Lauren classification | Intestinal type (n=176) | T/T | 1.00 (ref.) | 0.59 (0.23, 1.52) | 0.60 (0.22, 1.58) | 0.85 (0.24, 1.47) | 2.54 (0.87, 7.40) |
|  |  | T/C+C/C | 0.89 (0.37, 2.14) | 1.33 (0.60, 2.98) | 1.51 (0.91, 2.50) | P=0.007 | P*=*0.087 |
|  |  | ORs (95% CI)^a^ for genotype within strata of HP infection status | 0.83 (0.33, 2.09) | 2.23 (1.23, 4.07) |  |  |  |
|  | Diffuse type  (n=100) | T/T | 1.00 (ref.) | 2.60 (0.57, 11.9) | 2.72 (0.59, 12.46) | 0.96 (−1.01, 2.93) | 1.29 (0.23, 7.23) |
|  |  | T/C+C/C | 1.09 (0.22, 5.49) | 3.66 (0.86, 15.63) | 3.57 (1.54, 8.25) | P=0.338 | P*=*0.775 |
|  |  | ORs (95% CI)^a^ for genotype within strata of HP infection status | 0.90 (0.17, 4.81) | 1.42 (0.77, 2.63) |  |  |  |
|  | Mixed type  (n=32) | T/T | 1.00 (ref.) | 0.81 (0.08, 8.38) | 0.39 (0.03, 5.03) | 1.24 (−0.59, 3.07) | 2.54 (0.19, 34.81) |
|  |  | T/C+C/C | 1.00 (0.10, 9.56) | 2.04 (0.26, 16.29) | 1.98 (0.62, 6.31) | P=0.183 | P*=*0.484 |
|  |  | ORs (95% CI)^a^ for genotype within strata of HP infection status | 0.87 (0.07, 11.35) | 2.53 (0.69, 9.19) |  |  |  |

^a^ Adjusted for age, sex, cumulative smoking amount, alcohol intake amount, body mass index, and education level.

^b^ Interaction on an additive scale

^c^ Interactions on a multiplicative scale

OR = odds ratio; RERI = relative excess risk due to interaction
